# Absence of Association between *N*-Acetyltransferase 2 Acetylator Status and Colorectal Cancer Susceptibility: Based on Evidence from 40 Studies

**DOI:** 10.1371/journal.pone.0032425

**Published:** 2012-03-05

**Authors:** Lou qian Zhang, Jian nong Zhou, Jun Wang, Guo dong Liang, Jing ying Li, Yi dan Zhu, Yun tao Su

**Affiliations:** 1 Colorectal and Anal Surgery Center, The Affiliated Jiangsu Cancer Hospital of Nanjing Medical University, Nanjing, China; 2 Department of Chemotherapy, Jiangsu Geriatric Institute, Jiangsu Official Hospital, Nanjing, China; 3 Department of Gastroenterology, Jiangsu Geriatric Institute, Jiangsu Official Hospital, Nanjing, China; 4 Department of Medical Oncology, The First Affiliated Hospital of Nanjing Medical University, Nanjing, China; National Cancer Institute, National Institutes of Health, United States of America

## Abstract

**Background and Objectives:**

*N*-Acetyltransferase (NAT) 2 is an important enzyme involved in the metabolism of different xenobiotics, including potential carcinogens, whose phenotypes were reported to be related to individual susceptibility to colorectal cancer (CRC). However, the results remain conflicting. To assess the relationship between *NAT2* phenotypes and CRC risk, we performed this meta-analysis.

**Methods:**

A comprehensive literature search was conducted to identify all case-control or cohort studies of NAT2 acetylator status on the susceptibility of CRC by searching of PubMed and EMBASE, up to May 20, 2011. Crude odds ratios (ORs) with 95% confidence intervals (CIs) were used to assess the association.

**Results:**

A total of over 40,000 subjects from 40 published literatures were identified by searching the databases. No significantly elevated CRC risk in individuals with NAT2 slow acetylators compared with fast acetylators was found when all studies pooled (OR = 0.95, 95% CI: 0.87–1.04, I^2^ = 52.6%). While three studies contributed to the source of heterogeneity were removed, there was still null result observed (OR = 0.96, 95% CI: 0.90–1.03, P = 0.17 for heterogeneity, I^2^ = 17.8%). In addition, we failed to detect any associations in the stratified analyses by race, sex, source of controls, smoking status, genotyping methods or tumor localization. No publication bias was observed in this study.

**Conclusions:**

This meta-analysis suggests that the *NAT2* phenotypes may not be associated with colorectal cancer development.

## Introduction

Colorectal cancer (CRC) is the third most common cancer among men and women in the U.S., and ranks third as a cause of cancer deaths [1]. The etiology of CRC is complex and multifactorial. Hereditary syndromes, such as familial adenomatous polyposis (FAP) and hereditary nonpolyposis colorectal cancer (HNPCC), account for less than 10% of all cases [2]. The majority of CRC is sporadic and thought to be caused by multiple factors, which include dietary and lifestyle habits and/or mild genetic predisposition [3]. As many experimental work and genetic epidemiological studies conducted, many risk factors associated with colorectal carcinogenesis are under spot light. There is considerable evidence in support of an association between tobacco smoke and colorectal cancer [4]. Another well-established risk factor for CRC is red meat and, particularly, processed meat [5]. One of the hypothesized mechanisms to explain an increased CRC risk with smoking and meat intake is through exposure to carcinogenic aromatic and heterocyclic amines (such as benzidine) [6]. The metabolic activation of both aromatic and heterocyclic amines is catalyzed by *N*-acetyltransferases (NAT) 1 and/or 2 that are coded by genes (*NAT1* and *NAT2*) which are highly polymorphic. The carcinogenic amines can be metabolized more or less efficiently in individuals depending on their *NAT* genotypes. The *NAT2* gene, which is located on chromosome 8p21.3–23.1, and encodes phase II xenobiotic metabolizing enzyme which plays an essential role in the metabolism of aromatic, heterocyclic amines and hydrazines via *N*-acetylation and O-acetylation [7]. It has been demonstrated that the variant alleles in *NAT2* result in slow clearance of carcinogenic amines [8] Thus, a role for *NAT2* acetylation polymorphism in individual risk to various cancers in which carcinogens exposure play an etiologic role is biologically plausible and has been the subject of numerous studies. The high frequency of the *NAT2* acetylation polymorphisms in human populations together with ubiquitous exposure to aromatic and heterocyclic amines suggest that *NAT2* acetylator genotypes are important modifier of human cancer susceptibility. So far, over sixty *NAT2* genetic variants have been identified in human being, in which NAT2*4 is the most common allele associated with rapid acetylation and has historically been designated “wildtype”. The *NAT2* alleles are regularly updated and listed at: http://www.louisville.edu/medschool/pharmacology/NAT.html by an international gene nomenclature committee. Detailed information on *NAT2* alleles is also provided in a supplemental file. To date, a number of epidemiological studies have investigated the potential role of *NAT2* polymorphisms in colorectal cancer development. However, the results were inconsistent rather than conclusive, probably due to the possible small effect of differential acetylator status on CRC risk or the relatively small sample size in individual studies. Therefore we performed a meta-analysis to get a more precise estimate of the relationship between *NAT2* phenotypes and colorectal cancer risk.

## Results

### Eligible studies

**Figure 1:**
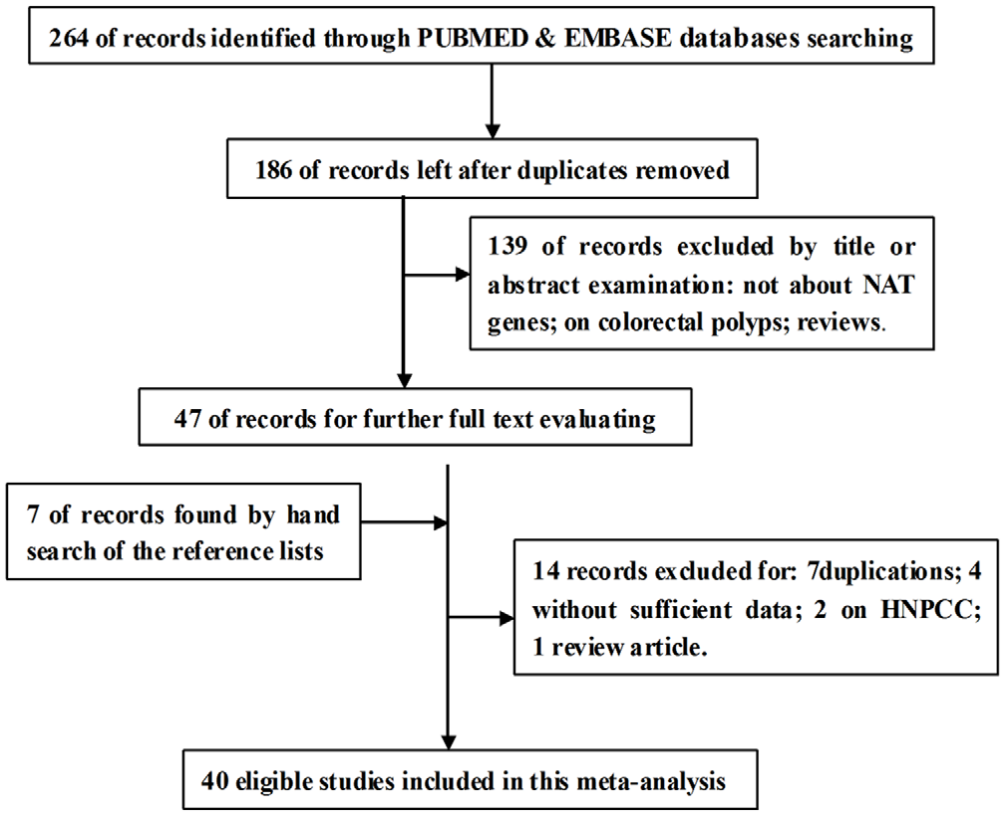
Flow chart indicates the inclusion and exclusion of studies.

A total of 186 potentially relevant articles were retrieved through electronic databases searching that met our criteria. After carefully reviewed the titles and abstracts, 139 articles were excluded for not about *NAT* genes or on colorectal polyps or reviews. The rest 47 relevant studies were obtained for further full text evaluating. Seven literatures were also found by hand search of the reference lists. After information extraction and discussing, 14 studies were further excluded (7 duplications, 4 without sufficient data, 2 on HNPCC and 1 review paper), resulting in 40 eligible studies with 13,896 CRC cases and 18,839 controls reporting the association between the *NAT2* acetylator phenotypes and CRC risk for this meta-analysis [9]–[48]. The study selection process is outlined in Figure 1. Table S1 lists the main characteristics of the eligible studies. Among them, 19 studies were conducted on Caucasians, 10 on Asians and 11 on mixed populations. Only one study by Butler *et al.* investigated African population [22]. Four of the 40 studies were hospital-based but a much larger proportion was population-based (90%), thus representing the general population. Half of the studies were matched at least one of the following confounding factors: age, sex, ethnicity, smoking, or meat consumption. The classic polymerase chain reaction-restriction fragment length polymorphism (PCR-RFLP) assay was used in twenty studies.

### Quantitative synthesis

**Figure 2:**
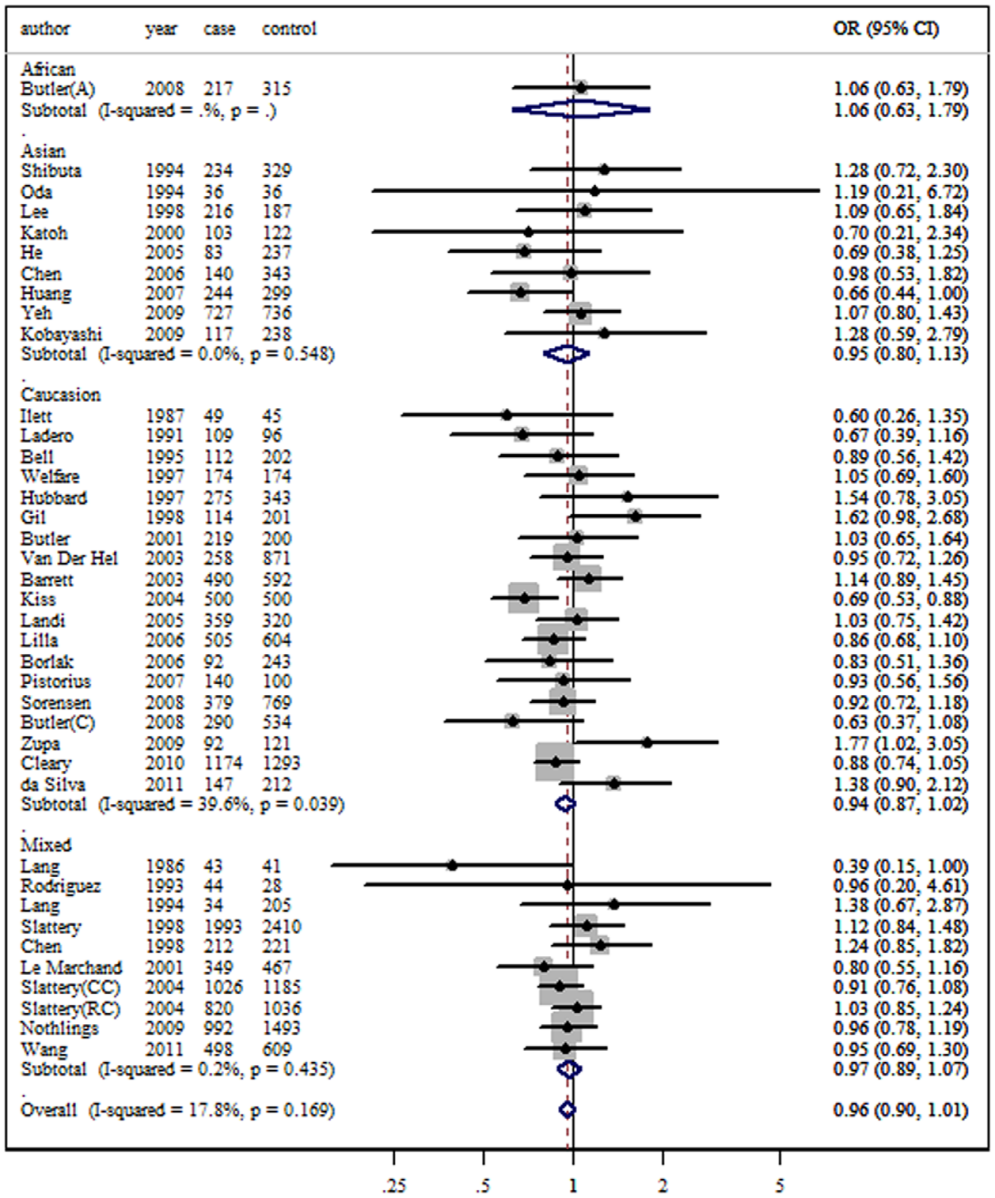
Forest plots of overall association between *NAT2* phenotypes and colorectal cancer risk (slow acetylation *versus* rapid acetylation genotypes).

The crude ORs were performed for slow versus rapid acetylation genotypes. Individuals with the *NAT2* slow phenotypes were not statistically significant associated with an increased risk to colorectal cancer compared with those carrying rapid phenotypes. The summary OR was 0.95 (95% CI: 0.87–1.04, P = 0.00 for heterogeneity, I^2^ = 52.6%). There was substantial heterogeneity among these studies. Herein, we explored the source of heterogeneity. When the three studies removed [23], [25], [36], the heterogeneity dropped sharply, but the summary estimate was not materially altered (OR = 0.96, 95% CI: 0.90–1.01, P = 0.17 for heterogeneity, I^2^ = 17.8%). Figure 2 shows the forest plot of overall comparison between slow and rapid acetylator phenotypes.

In the subgroup analysis by ethnicity, no increased risk was found for either Caucasians (OR = 0.94, 95% CI = 0.87–1.02) or Asians (OR = 0.95, 95% CI = 0.80–1.03). So dose mixed populations (OR = 0.97, 95% CI = 0.89–1.07). In addition, significant associated increasing risk was not detected in different source of controls (for population based controls: OR = 0.98, 95% CI = 0.89–1.07; for hospital based controls: OR = 0.79, 95% CI = 0.53–1.16). When stratified by genotyping methods, gender, smoking status and tumor site, similarly, few significant associations was found for all of these subgroup analyses. The main results of this meta-analysis and the heterogeneity tests are shown in Table 1.

**Table 1:** Main results of overall and subgroups in the meta-analysis.

Overall and subgroups analyses	No. of studies	OR	95% CI	Heterogeneity	
				P value	I^2^ (%)
*Total*	42	0.95	0.87–1.04	<0.001	52.6
*Total* *	39	0.96	0.90–1.01	0.17	17.8
*Ethnicity*					
Asian	9	0.95	0.80–1.03	0.55	0.00
Caucasian	19	0.94	0.87–1.02	0.04	39.6
Mixed	10	0.97	0.89–1.07	0.44	0.20
*Source of controls*					
Population based	37	0.98	0.89–1.07	0.001	52.8
Hospital based	4	0.79	0.53–1.16	0.44	0.00
*Genotyping methods*					
PCR-RFLP	20	0.98	0.87–1.10	0.04	39.8
PCR-RFLP/(AS)-PCR	3	0.81	0.57–1.16	0.39	0.00
F-based melting curve	2	0.87	0.70–1.09	0.78	0.00
TaqMan	4	0.72	0.46–1.12	<0.001	89.0
*Gender*					
Male	6	1.16	0.95–1.42	0.94	0.00
Female	6	1.03	0.74–1.42	0.04	57.1
*Smoking status*					
Never smoke	5	0.93	0.66–1.32	0.01	69.8
Ever smoke	6	0.89	0.69–1.15	0.07	51.9
*Tumor site*					
Colon cancer	3	0.91	0.80–1.05	0.86	0.00
Rectal cancer	3	1.07	0.92–1.24	0.77	0.00

* Three studies excluded for exploring the source of heterogeneity. [23], [25], [36], confidence interval, CI.

### Sensitivity analyses

In sensitivity analysis, individual study was sequentially removed each time, and the results suggested that no individual study obviously affected the summary OR, which indicated that our results were statistically robust. However, sensitivity analyses also indicated that three separate studies by Mahid *et al.*, Yoshida *et al.* and Tiemersma *et al.* were the main origin of the heterogeneity. After excluding them, heterogeneity test was below statically significant.

### Publication bias

**Figure 3:**
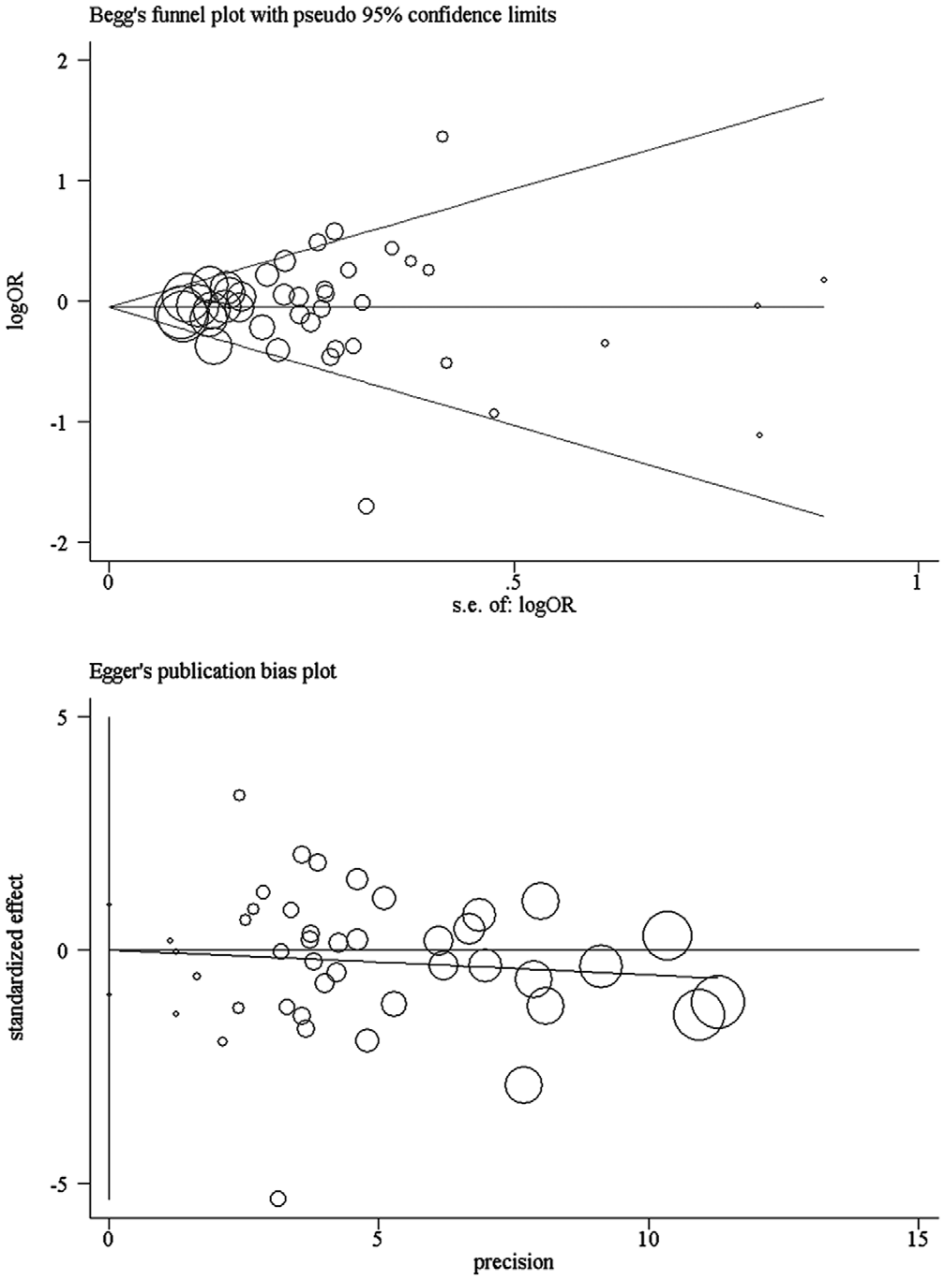
Funnel plots of Begg's and Egger's were used to detect publication bias on overall estimate. No significant publication bias was found. Each point represents an individual study for the indicated association.

As shown in Figure 3, the shape of the funnel plot seemed symmetrical, suggesting the absence of publication bias. Then, the Egger's test was adopted to provide statistical evidence of funnel plot symmetry. The result still did not suggest any evidence of publication bias (P = 0.89).

## Discussion

This meta-analysis based on 40 studies involving over 40,000 subjects indicates that lack of sufficient evidence supporting the notion of *NAT2* phenotypes correlating with the risk of colorectal cancer. In addition, stratified analyses according to ethnicity, source of controls, phenotyping/genotyping methods, gender, smoking status and tumor site also indicate that *NAT2* acetylator status is not associated with CRC predisposition based on the currently available data. As we know, this is the largest meta-analysis of the comprehensive assessment for the relationship between *NAT2* phenotypes and CRC risk. The previous meta-analysis didn't support the hypothesis that *NAT2* alone is an important risk factor for colorectal cancer and suggests that *NAT2* rapid acetylation status has no specific effect on the risk of developing colorectal cancer [49]. Though there are some limits containing in the meta-analysis (such as misclassification in case group.), the conclusion is in consistent with ours.

Studies by meta-analysis have shown that *NAT2* genetic polymorphisms are associated with some malignancies. Reports by Simon Sanderson *et al.* supported the evidence that a contribution of *NAT2* slow acetylation status alone to bladder carcinogenesis and provided support for a *NAT2*-smoking interaction [50]. However, results from other meta-analyses on breast, lung and stomach carcinogenesis suggested that there is no overall association between the *NAT2* slow or rapid acetylation phenotype [51]–[53]. The role of *NAT2* genetic polymorphisms in the development of CRC may be modified by confounding factors. Processed meat and smoking interact with *N*-acetyltransferases efficiency on colorectal cancer risk have received a great deal of attention in some studies. Moreover, an enhanced association between smoking and colorectal cancer risk in subjects with the *NAT2* rapid genotype was detected and supported a role for *NAT2* and tobacco smoke heterocyclic amines in the etiology of colorectal cancer [19]. Meanwhile, detoxification of polycyclic aromatic hydrocarbons may involve many other genes that might influence the action of *NAT2* examined. Other genes, including other *CYP*s, *GST*s, *NAT1*, and the aromatic hydrocarbon receptor gene, which positively regulates inducible expression of aromatic hydrocarbon hydroxylase, may further define genetic susceptibility to the exposure of carcinogenic amines [32]. Even if heterocyclic amines are a causal factor for CRC, other enzymes are involved in the activation and detoxification of such compounds, thus accounting only for NAT2 provides only a partial picture of the whole pathway.

Since multiple mechanisms for reductions in NAT2 activity are associated with various combinations of variants that make up *NAT2* alleles, the ability to distinguish among multiple acetylator phenotypes is complex and a function of the sensitivity and specificity of the genotyping method. Furthermore, phenotypes are influenced by a number of factors including diet, disease, and drug therapy. Depending upon the probe drug and analytical method used, acetylation phenotypes often exhibit overlap due to numerous genetic and/or environmental factors, including the large number and diversity of *NAT2* genotypes present in human populations (reviewed in [54]). Thus, the nondifferential misclassification would likely bias the results to the null.

Some limitations of this meta-analysis should be mentioned. First of all, ethnic differences in *NAT2* allelic frequencies are quite striking and should be cautious when interpreting the results. e.g. the prevalence of NAT2 slow acetylators in European whites is approximately 56% and about 11% among Asians [55]. The difference can lead to bias in choosing control groups and masking the effect of NAT2 acetylator in molecular epidemiologic studies. Secondly, there was significant heterogeneity between the studies. We explored the source of heterogeneity and found three studies could possibly explain the origin of observed heterogeneity. The cause of the heterogeneity may be partially explained by the ethnic diversity (two studies select mixed ethnic control groups [25], [36]). However, the systemic result was not affected after the exclusion. Thirdly, the sample size is still relatively small for some stratified analyses, might fail to detect small effect of *NAT2* phenotypes on colorectal cancer risk in specific stratification. Although no correlation between *NAT2* phenotypes and CRC risk was found pooling the data from 6 studies which stratified smoking status, a precise measurement of exposure to carcinogens may be helpful to understand the biological mechanisms when genes involved in metabolic pathways are assessed. Lastly, the results were based on unadjusted estimates, while a more precise analysis should be conducted if all individual raw data were available, which would allow for an adjustment estimate.

In spite of these limitations, this meta-analysis had several strengths. First, it is the largest number of cases and controls were pooled to date, which substantially increased the statistical power of the analysis. Second, no publication biases were detected, indicating that summary results may be unbiased. Third, in the sensitivity analysis, no individual study affected the pooled OR which indicated that our results were statistically robust and trustworthy.

In conclusion, the meta-analysis failed to detect a significant association between *NAT2* phenotypes and predisposition to colorectal cancer. However, CRC is a multifactor and multiprocessing disease that resulted from complex interactions between gene–gene and gene–environment. Therefore, further large and well-designed epidemiological studies with considering potential interactions and more precise measurement of exposure to carcinogens are needed.

## Methods

### Identification and eligibility of relevant studies

We searched for relevant papers published before May 20, 2011 using the electronic PubMed and EMBASE databases with the following terms and their combinations: ‘*NAT2*’ or ‘*N*-acetyltransferase 2’, ‘colon cancer’, ‘rectal cancer’, ‘colorectal cancer’ and ‘polymorphism’ or ‘variant’. No language restriction was imposed. References of the retrieved articles were also reviewed for additional studies. We included all the case–control and cohort studies that reported the association between *NAT2* polymorphisms and colorectal cancer risk with sufficient data for estimating an odds ratio (OR) and their 95% confidence intervals (CIs). Abstracts, unpublished data were not considered. Investigations in subjects with family cancer risks or cancer-prone disposition were excluded. Besides, when the same study population was included by more than one article, we selected the study that included the largest number of individuals.

### Data extraction

We extracted the following information from each study: first author's surname, year of publication, country of origin, ethnicity, phenotyping/genotyping information, source of control groups (population-based, hospital-based or mixed controls), matching criteria and number of different genotypes in all subjects. The ethnicity of studies was categorized as Asians, Caucasians, African or Mixed. We defined carriers with at least one of the high-activity alleles as rapid acetylators, in accordance with the definition in most studies, whereas individuals carrying two low-activity alleles were considered as slow acetylators. All the data were extracted separately by two authors (Zhang LQ and Wang J), and the disagreement was solved by discussing.

### Statistical analysis

The meta-analysis was performed to estimate the risk of colorectal cancer associated with *NAT2* slow/rapid acetylation polymorphisms. Crude ORs with 95% CIs were calculated using raw data, according to the method of Woolf B. [56]. In addition to the comparison among overall subjects, we also performed stratified analyses by ethnicity, genotyping method, source of controls, gender, tumor localization and smoking status. We investigated the between-study heterogeneity by using the Cochran's Q-test and estimating I^2^, respectively. And the heterogeneity was considered significant, if P<0.10 for Q-test [57]. A p-value >0.10 for the Q-test indicated a lack of heterogeneity across the studies, and a fixed-effect model (the Mantel–Haenszel method) [58] was used, otherwise a random-effect model (the DerSimonian and Laird method) [59] was used. One-way sensitivity analysis was performed to assess the stability of the results, namely, a single study in the meta-analysis was deleted each time to reflect the influence of the individual data set to the pooled OR. Funnel plots and the Egger's test were used to examine the influence of publication bias (linear regression analysis) [60]. All analyses were conducted using Stata software (version 11.0; Stata Corp LP, College Station, TX). All the p-values were two-sided.

## Supporting Information

Table S1 Main characteristics of studies included in the meta-analysis.
(DOC)
