# Supplementary material for: Absence of Association between N-Acetyltransferase 2 Acetylator Status and Colorectal Cancer Susceptibility: Based on Evidence from 40 Studies
Source: PLoS One. 2012 Mar 5;7(3):e32425. doi: 10.1371/journal.pone.0032425 (PMC3293792; doi:10.1371/journal.pone.0032425)
Supplement: Table S1 — Main characteristics of studies included in the meta-analysis. (DOC) [file pone.0032425.s001.doc]

| **Table 1 Main characteristics of studies included in the meta-analysis.** | | | | | | | | |
| --- | --- | --- | --- | --- | --- | --- | --- | --- |
| First author | Year | Country | Ethnicity | Source of controls | Drug-based Phenotyping /Genotyping methods | cases | controls | Matching criteria |
| Wang[19] | 2011 | USA | Mixed | Population based | TaqMan | 498 | 609 | sex, ethnicity and age(±2 years) |
| da Silva[20] | 2011 | Brazil | Caucasian | Population based | PCR-RFLP | 147 | 212 | － |
| Cleary[21] | 2010 | Canada | Caucasian | Population based | TaqMan | 1174 | 1293 | sex and age(±5 year) |
| Zupa[22] | 2009 | Italy | Caucasian | Population based | PCR-RFLP | 92 | 121 | － |
| Yeh[23] | 2009 | Taiwan | Asian | Population based | PCR-RFLP/PE-DHPLC | 727 | 736 | sex and age |
| Nothlings[24] | 2009 | USA | Mixed | Population based | Taqman/Sequence Detection System | 992 | 1493 | sex, ethnicity/race and age |
| Kobayashi[25] | 2009 | Japan | Asian | Population based | MassARRAY | 117 | 238 | － |
| Sorensen[26] | 2008 | Denmark | Caucasian | Population based | Taqman/Sequence Detection System | 379 | 769 |  |
| Butler^A^[27] | 2008 | USA | African | Population based | PCR-RFLP/(AS)-PCR | 217 | 315 | by sex, ethnicity and age |
| Butler^C^[27] | 2008 | USA | Caucasian | Population based | PCR-RFLP/(AS)-PCR | 290 | 534 | by sex, ethnicity and age |
| Yoshida[28] | 2007 | Japan | Asian | Population based | PCR-RFLP | 66 | 121 | － |
| Pistorius[29] | 2007 | Germany | Caucasian | Population based | RT-PCR+F-based melting curve | 140 | 100 | － |
| Mahid[30] | 2007 | USA | Mixed | Population based | TaqMan | 122 | 222 | － |
| Huang[31] | 2007 | Taiwan | Asian | Population based | PCR-RFLP | 244 | 299 | age and gender |
| Lilla[32] | 2006 | Germany | Caucasian | Population based | RT-PCR+F-based melting curve | 505 | 604 | sex and age(±5 year) |
| Borlak[33] | 2006 | Germany | Caucasian | Population based | PCR-RFLP | 92 | 243 | － |
| Landi[34] | 2005 | Italy | Caucasian | Population based | Sequence Detection System | 359 | 320 | － |
| He[35] | 2005 | China | Asian | Population based | PCR-RFLP | 83 | 237 | － |
| Chen[36] | 2006 | China | Asian | Population based | PCR-RFLP | 140 | 343 | － |
| Slattery^CC^[37] | 2004 | USA | Mixed | Population based | PCR-RFLP | 1026 | 1185 | sex and age(±5 year) |
| Slattery^RC^[37] | 2004 | USA | Mixed | Population based | PCR-RFLP | 820 | 1036 | sex and age(±5 year) |
| Kiss[38] | 2004 | Hungary | Caucasian | Mixed | PCR-RFLP | 500 | 500 | sex, age, smoking, meat consumption |
| Van Der Hel[39] | 2003 | Netherlands | Caucasian | Population based | PCR-RFLP | 258 | 871 | － |
| Barrett[40], | 2003 | UK | Caucasian | Population based | TaqMan | 490 | 592 | sex and age |
| Tiemersma[41] | 2002 | Netherlands | Mixed | Population based | allele-speciﬁc hybridization assay | 102 | 539 | sex and age |
| Le Marchand[42] | 2001 | USA | Mixed | Population based | PCR-RFLP | 349 | 467 | by sex, ethnicity and age(±2 years) |
| Butler[43] | 2001 | Australia | Caucasian | Population based | PCR-RFLP | 219 | 200 | gender |
| Katoh[44] | 2000 | Japan | Asian | Population based | PCR-RFLP/(AS)-PCR | 103 | 122 | － |
| Slattery[45] | 1998 | USA | Mixed | Population based | oligonucleotide ligation assay | 1993 | 2410 | sex and age |
| Lee[46] | 1998 | Singapore | Asian | Population based | allele-speciﬁc PCR | 216 | 187 | － |
| Gil[[50] | 1998 | Portugal | Caucasian | Population based | PCR-RFLP | 114 | 201 | － |
| Chen[47] | 1998 | USA | Mixed | Population based | PCR-RFLP | 212 | 221 | age(±1year),smoking |
| Welfare[48] | 1997 | UK | Caucasian | Population based | PCR-RFLP | 174 | 174 | age and sex |
| Hubbard[49] | 1997 | UK | Caucasian | Population based | PCR-RFLP | 275 | 343 | sex |
| Bell[51] | 1995 | UK | Caucasian | Hospital based | PCR-RFLP | 112 | 202 | － |
| Shibuta[14] | 1994 | Japan | Asian | Population based | PCR-RFLP | 234 | 329 | － |
| Oda[15] | 1994 | Japan | Asian | Hospital based | Southern blotting | 36 | 36 | age |
| Lang[16] | 1994 | USA | Mixed | Population based | Caffeine for phenotype | 34 | 205 | － |
| Rodriguez[17] | 1993 | USA | Mixed | Hospital based | PCR-RFLP | 44 | 28 | － |
| Ladero[18] | 1991 | Spain | Caucasian | Population based | sulfamethazine phenotype | 109 | 96 | age |
| Ilett[52] | 1987 | Australia | Caucasian | Population based | sulfamethazine phenotype | 49 | 45 | － |
| Lang[53] | 1986 | USA | Mixed | Hospital based | sulfamethazine phenotype | 43 | 41 | － |
| ^A^ African, ^C^ Caucasian, ^CC^ Colon Cancer, ^RC^ Rectal Cancer. PCR-RFLP: polymerase chain reaction-restriction fragment length polymorphism, RT-PCR: real time PCR | | | | | | | | |
| PE-DHPLC: primer extension and denaturing high performance liquid chromatography, F-based: Fluorescence-based. | | | | | | | | |
